# Changes in the Monocytic Subsets CD14^dim^CD16^+^ and CD14^++^CD16^−^ in Chronic Systolic Heart Failure Patients

**DOI:** 10.1155/2012/616384

**Published:** 2012-11-27

**Authors:** Offer Amir, Ilia Spivak, Idit Lavi, Michal Amit Rahat

**Affiliations:** ^1^Heart Failure Center, Division of Cardiology, Lady Davis Carmel Medical Center and the Ruth and Bruce Rappaport Faculty of Medicine, Technion – Israel Institute of Technology, 34362 Haifa, Israel; ^2^Immunology Research Unit, Lady Davis Carmel Medical Center and the Ruth and Bruce Rappaport Faculty of Medicine, Technion – Israel Institute of Technology, 34362 Haifa, Israel; ^3^Department of Community Medicine and Epidemiology, Lady Davis Carmel Medical Center and the Ruth and Bruce Rappaport Faculty of Medicine, Technion – Israel Institute of Technology, 34362 Haifa, Israel

## Abstract

Different monocytic subsets are important in inflammation and tissue remodelling, but although heart failure (HF) is associated with local and systemic inflammation, their roles in HF are yet unknown. We recruited 59 chronic systolic HF patients (aged 58 ± 13 years, 45 males and 14 females) and 29 age-matched controls with no pervious heart disease. Compared to the controls, we found no change in the distribution of the CD14^+^CD16^+^ monocytic subset, whereas the classical CD14^++^CD16^−^ subset was decreased by 11% (*P* < 0.001), and the nonclassical CD14^dim^CD16^+^ subset was expanded by 4% (*P* < 0.001) in HF patients and was inversely associated with severe HF (*P* = 0.015), as assessed by increased end-diastolic dimension (EDD). Compared to the control group, serum TNF*α*, IL-1*β*, IL-10, and IL-13 levels were significantly elevated in the HF patients. Specifically, IL-13 levels were positively correlated to the CD1CD14^dim^CD16^+^ monocytic subset (*r* = 0.277, *P* = 0.017), and intracellular staining of IL-13 demonstrated that some of these monocytes produce the cytokine in HF patients, but not in the controls. We suggest that the inverse association between EDD values and the expansion of CD14^dim^CD16^+^ monocytes that can produce IL-13 could be explained as a measure to counterbalance adverse remodelling, which is a central process in HF.

## 1. Introduction

Inflammation plays an important role in the pathogenesis of heart failure (HF) and exerts an effect on its prognosis. Involvement of inflammatory mediators (e.g., cytokines), inflammatory markers [1], and oxidative stress [2–4] is known in HF and is associated with immune/inflammatory activation, myocardial hypertrophy, adverse myocardial remodelling, and increased mortality [5, 6]. Increased circulatory levels of both proinflammatory and anti-inflammatory cytokines, such as IL-6, TNF*α*, and IL-10, correlate with HF progression, severity, and increased mortality [6, 7]. Among leukocytes, activated monocytes and macrophages are considered a major source of both pro- and anti-inflammatory cytokines.

Although monocytes represent only 5–10% of peripheral blood leukocytes in humans, they play a major role in inflammatory processes and in tissue remodelling. This is due to their ability to phagocytose microorganisms, their products, or endogenous danger-associated molecular patterns (DAMPs) and process and present them to T cells to initiate an adaptive immune response. They also produce reactive oxygen and nitrogen species and secrete myriad of cytokines and growth factors in response to the stimulation. In fact, monocytes are main producers of proinflammatory and some anti-inflammatory cytokines, including TNF*α*, IL-6, and IL-10 [8]. Since it is not likely that so many diverse functions are carried out by the same cell, it was suggested that monocytes are a heterogeneous population of cells, each with distinct phenotypes and functions [8, 9].

The main markers used to distinguish between humans monocyte subsets are CD14 (part of the lipopolysaccharide receptor) and CD16 (Fc*γ*RIII). These markers define three distinct subsets: classical monocytes that express high levels of CD14 and no CD16 (CD14^++^CD16^−^, also termed CD14^+^CD16^−^), intermediate monocytes that express intermediate levels of CD14 and CD16 (CD14^++^CD16^+^ or CD14^+^CD16^+^), and nonclassical monocytes that express very low levels of CD14 and high levels of CD16 (CD14^dim^CD16^+^ or CD14^−^CD16^+^) [8, 10]. The two CD16^+^ subsets were shown to expand in many inflammatory conditions (e.g., cancer, sepsis, and stroke), whereas the CD14^++^CD16^−^ subsets remained unchanged or even decreased [8, 11–13]. However, the function of all these subsets as cells secreting either pro- or anti-inflammatory cytokines is still controversial. For example, sorted monocytes subsets that were stimulated *ex vivo* with LPS or zymosan, but not with *S. aureus*, increased IL-10 secretion from CD14^+^CD16^+^ cells, whereas LPS-stimulated CD14^dim^CD16^+^ cells showed increased secretion of TNF*α* [11]. In a different study, CD14^dim^CD16^+^ cells showed patrolling characteristics with weak ability for phagocytosis and low production of ROS and cytokines when challenged by bacterial ligands of toll-like receptors but secreted high amounts of the proinflammatory TNF*α* and IL-1*β* cytokines upon stimulation with viral ligands or nucleic acids [14]. Thus, it seems that the monocytic subsets cannot be simply defined as pro- or anti-inflammatory, and their functions may depend on the nature of the stimulus.

Involvement of different monocytes and macrophages subsets has already been described in acute myocardial infarction (AMI), in humans [15, 16], and in a mouse model [17], and the different kinetics of their recruitment to the heart as well as their different receptor expression suggested that they have different roles in healing and remodelling of the myocardium. Accordingly, the purpose of the current study was to characterize possible changes in the distribution of monocyte subsets in patients with chronic systolic HF, and to further explore the potential impact of these subsets on specific key inflammatory cytokines as well as on clinical parameters of HF.

## 2. Materials and Methods

### 2.1. Patients

We recruited 59 patients with systolic HF from our out patients clinic: 45 males and 14 females. For comparison, we also recruited a group of 29 age-matched controls: 15 males and 14 females. 

Inclusion criteria for HF patients were stage C, New York Heart Association class (NYHA) of 2–4, chronic systolic HF (left ventricular ejection fraction <40% per echocardiogram), and ages 18–90 years. On recruitment, HF patients had to be in their usual clinical stable status with no recent cardiac decompensation. All patients were treated according to the AHA/ACC guidelines, and their characteristics are summarized in Table 1.

Exclusion criteria for the HF group were recent (≤1 month) admission for acute heart failure or acute coronary syndrome, or haemodialysis treatment or known systemic inflammatory disease or recent (<1 month) febrile illness. 

The control group consisted of volunteers that were individually evaluated by a board certified cardiologist based on a detailed personal interview of medical history, review of available medical records, and medical treatments. Exclusion criteria for these volunteers were prior history of coronary/structural myocardial disease, systemic inflammatory disease, or a recent febrile illness (<1 month). Of note, history and/or treatment for diabetes mellitus/hypertension were not considered as exclusion criteria for the control group.

In all study participants, (59 HF patients and 29 controls), a single blood sample was drawn in the morning hours for analysis of both monocytes subsets (whole blood) and cytokines (serum sample). 

The study conforms to the principles outlined in the Declaration of Helsinki and was approved by the local Helsinki committee of Lady Davis Carmel Medical Centre, and all participants signed a written informed consent prior to their inclusion in the study. 

### 2.2. Monocyte Phenotyping

EDTA anticoagulated whole blood was collected from controls and HF patients. To avoid the activation of monocytes whole blood was used, and red blood cells were lysed with Uti-Lyse reagent (DAKO, Carpinteria, CA, USA) followed by two washes with PBS. Cells were resuspended in RPMI 1640 with 1% FCS and stained with fluorescently labelled monoclonal antibodies (PerCP anti-human CD16 clone 3G8, BioLegend, San Diego, CA, USA; Allophycocyanin (APC)-Alexa Fluor 750 anti-human CD14 clone 61D3, APC anti-human HLA-DR clone LN3, and appropriate isotype controls, eBioscience, San Diego, CA, USA) for 15 minutes at room temperature followed by an additional wash with PBS. In some samples, monocyte subsets were stained for intracellular IL-13 expression using permeabilization buffer and PE-anti-human IL-13 clone 32007 or its isotype control (R&D systems, Minneapolis, MN, USA). After washing, cells were fixed in PBS with 0.1% formaldehyde and were analysed using a LSR-II flow cytometer (BD, Bedford, MA). We used both compensation beads and isotype controls to determine the nonexpressing CD14 and CD16 cells. We first gated on all monocytes and granulocytes by their side and forward scattering and then further gated on the HLA-DR^+^ monocytes, as described in [18] to ensure the exclusion of CD16^+^ NK cells. The three different monocyte subpopulations were defined according to their expression of CD14 and CD16, as was described before [9, 11, 14, 18]. 

### 2.3. Cytokines

Serum cytokines from both patients and controls were measured with commercial ELISA kits for TNF*α*, IL-1*β*, IL-10, IL-13, and TGF*β* (eBioscience, San Diego, CA, USA) according to the manufacturer's instructions.

### 2.4. Statistical Analyses

Data were analysed by both the GraphPad Prism 5 program and the SPSS statistical package (version 18). Comparisons of two experimental groups were carried out using the nonparametric Mann-Whitney test. As many of the cytokines showed very low levels, we transformed the data into categorical variables according to the calculated median values of both controls and HF patients, and then the Pearson chi-square test was used to determine the association between different cytokines and HF. Logistic regression models were used to assess the association between HF and monocyte subsets and specific cytokines, controlled by age and gender. Odds ratios and 95% confidence interval were estimated from the models. Receiver operating characteristic (ROC) curves were used to evaluate the performance of each of the cytokines in classified HF patients. To calculate the correlation between monocyte subsets and the cytokines we used the Pearson or Spearman correlation analyses as appropriate. All *P* values were two-sided, and statistical significance was defined as *P* < 0.05.

## 3. Results

### 3.1. Monocyte Subsets

**Figure 1:**
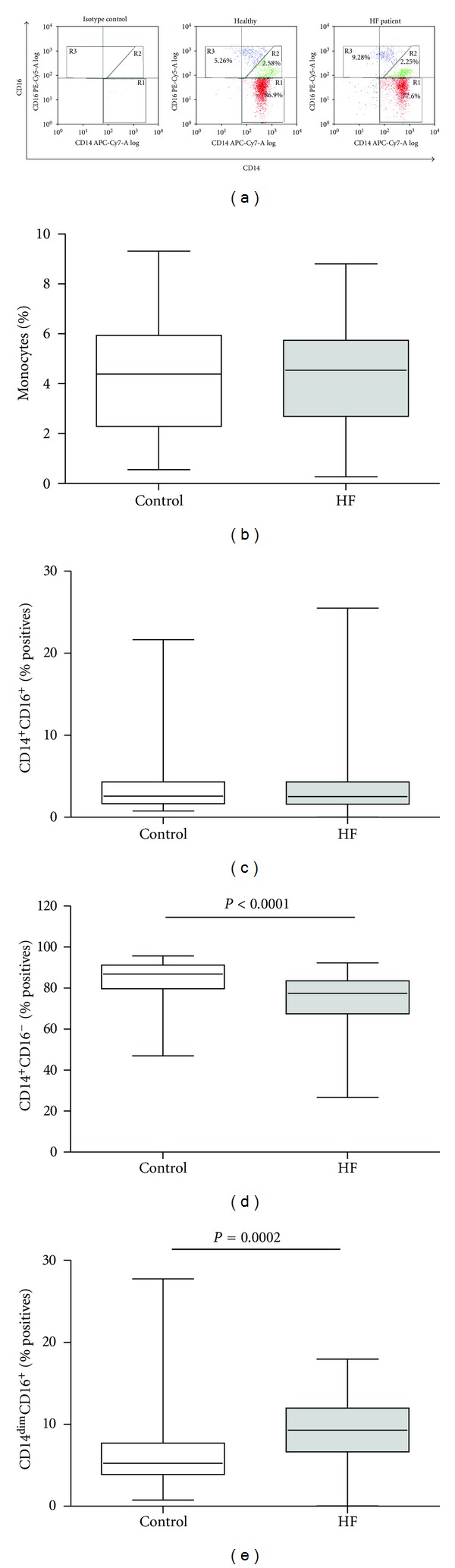
Characterization of monocyte subsets in HF patients and controls. (a) Representative flow cytometry dot plots of monocyte subsets in a healthy control and an HF patient, including the median values of each subset. Monocytes were gated by their side and forward scattering, and (b) their percentage from total blood leukocytes was determined; monocytes that were positive for HLA-DR expression were separated into three subsets according to their expression of CD14 and CD16, and their percentage of the total monocytes was determined in (c) CD14^+^CD16^+^ monocytes (gated R2 in (a), the green subpopulation), (d) CD14^++^CD16^−^ monocytes (gated R1 in (a), the red subpopulation), and (e) CD14^dim^CD16^+^ monocytes (gated R3 in (a), the blue subpopulation).

There was no significant difference in total monocyte percentage between the HF and the control groups (Figure 1(b)). In order to detect more specific changes, we measured differences in specific monocytes subsets, by using flow cytometry analysis based on the recently accepted division of CD14 and CD16 expressing monocytes [8, 10]. Monocytes were separated into three subsets (Figure 1(a)): classical CD14^++^CD16^−^ (gated in R1 in red), intermediate CD14^+^CD16^+^ (R2 in green), and nonclassical CD14^dim^CD16^+^ (R3 in blue) subsets. The majority of circulating monocytes were CD14^++^CD16^−^ (above 70% of all monocytes), but this subset was reduced in HF patients relative to the healthy controls by 11%, (84.3 ± 1.9, median 86.9 versus 73.5 ± 1.8, median 77.5, *P* < 0.0001, Figure 1(d)). In contrast, the nonclassical CD14^dim^CD16^+^ subset was significantly expanded in HF patients (mean 9.3 ± 0.5%, median 9.28 versus mean 6.5 ± 0.98%, median 5.26, *P* < 0.0002, Figure 1(e)). The CD14^+^CD16^+^ subset, however, consisted of only 3-4% of the circulating monocytes and showed no difference between controls and HF patients (3.9 ± 0.76, median 2.58 versus 3.6 ± 0.55, median 2.52, Figure 1(c)). Association of each monocyte subset to the presence of HF disease was evaluated using a logistic regression model and was found significant only for the expansion of the CD14^dim^CD16^+^ and reduction of the CD14^++^CD16^−^ subsets (Table 2).

Looking at the HF group, we further investigated the possible association between the expanded CD14^dim^CD16^+^ or the reduced CD14^++^CD16^−^ subsets and several parameters of HF severity, including left ventricular end-diastolic dimension (EDD), left ventricular ejection fraction (LVEF), and New York Heart Association (NYHA) class on the day of recruitment (Table 3). Because of the obvious homogeneity of the HF group (symptomatic, systolic, HF patients), we divided the monocytic subsets and the clinical parameters according to their medians. The CD14^dim^CD16^+^ subset was significantly associated only with inverse EDD values, consistent with less-adverse myocardial remodelling, whereas the CD14^++^CD16^−^ subset was not significantly associated with any of the clinical parameters. Similar analysis performed on the CD14^++^CD16^−^ subset revealed no significant association with these HF parameters. In order to further investigate whether the presence of CD14^++^CD16^−^ impacts the association between CD14^dim^CD16^+^ and cardiomegaly, we performed chi-square multiple comparisons tests (where a significant *P* value is considered only <0.01), by dividing the patients into four subgroups, based on combinations of low and high median values for CD14^++^CD16^−^ and CD14^dim^CD16^+^. The only significant difference in the association to decreased EDD values was found in the high CD14^++^CD16^−^/high CD14^dim^CD16^+^ group, compared to the high CD14^++^CD16^−^/low CD14^dim^CD16^+^ (*P* = 0.001). All other comparisons were not significant, including the comparison between low CD14^++^CD16^−^/high CD14^dim^CD16^+^ and high CD14^++^CD16^−^/high CD14^dim^CD16^+^ subsets (*P* = 0.07). Additionally, we performed a chi-square goodness of fit test, in which we compared the distribution of high CD14^++^CD16^−^/high CD14^dim^CD16^+^ to the distribution of the low and high EDD in the high CD14^dim^CD16^+^ group (without CD14^++^CD16^−^ subgroup division, Table 3), but no difference was found (*P* = 0.16). Collectively, this means that the only important parameter that affects EDD values is the CD14^dim^CD16^+^ subset, and the CD14^++^CD16^−^ values have no effect on the associative protective effect of CD^dim^CD16^+^ on the heart size.

### 3.2. Cytokine Expression

We compared the concentrations of 5 key cytokines (TNF*α*, IL-1*β*, IL-10, IL-13, and TGF*β*) in the sera of controls and HF patients (Table 4). As these cytokines do not have clear cutoff values, we determined cutoff values for each cytokine according to its median value and receiver-operator characteristics (ROC) curve analysis. As demonstrated in Table 4, with the exception of TGF*β*, all 4 cytokines were significantly increased in the HF group, compared to the control group.

### 3.3. Association between Monocyte Subsets and Cytokines

**Figure 2:**
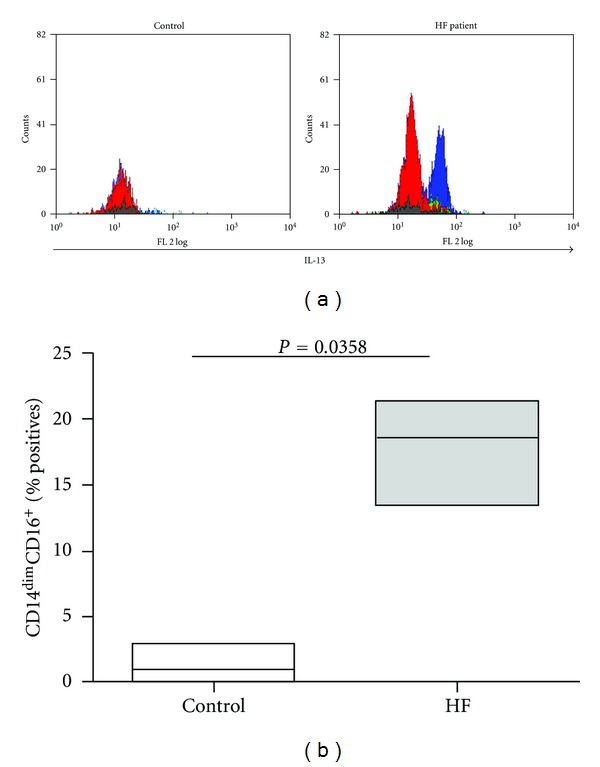
Production of IL-13 by each of the monocyte subsets. (a) Representative flow cytometry histograms of IL-13 producing monocyte subsets in a healthy control and an HF patient. Each of the three monocytes subsets was gated as described in Figure 1, and their respective ability to express IL-13 was evaluated by intracellular staining for the cytokine (*n* = 5). Grey histogram, isotype control for IL-13; red histogram, the CD14^++^CD16^−^ classical subset; green histogram, the CD14^+^CD16^+^ subset; blue histogram, the CD14^dim^CD16^+^ nonclassical subset. (b) Their percentage (median values) from the CD14^dim^CD16^+^ monocytes was determined.

Since monocytes may influence the inflammatory response by secreting both pro- and anti-inflammatory cytokines, we assessed the possible correlation between the two monocytic subsets (CD14^dim^CD16^+^ and CD14^++^CD16^−^) and the investigated serum cytokines (TNF*α*, IL-1*β*, IL-10, TGF*β*, and IL-13) (Table 5). TNF*α*, IL-1*β*, and IL-13 were significantly but negatively linked with the classically activated CD14^++^CD16^−^. In contrast, only IL-13 was positively correlated with the CD14^dim^CD16^+^ subset. To further explore the relationship between the nonclassical CD14^dim^CD16^+^ and IL-13, we performed intracellular staining of the monocytes with anti-IL-13 and gated each of the three monocyte subsets defined in Figure 1 to observe their respective ability to produce IL-13.  Figure 2 shows that healthy controls did not produce IL-13 (only 1.1 ± 0.5%-positive cells), whereas in HF patients some of the CD14^dim^CD16^+^ monocytes (17.7 ± 2.3% positive cells, the blue histogram) shifted to the right and clearly expressed intracellular IL-13.

## 4. Discussion

We found in our current work that, comparing to noncardiac volunteers, chronic systolic HF patients demonstrated significant changes in the distribution of their monocyte subsets. These changes lead to higher serum IL-13 levels and were inversely linked with increased size of the failing heart.

Inflammation has long been associated with HF, with disease progression and adverse outcome [7, 19]. However, the role of the cells responsible for these effects has not yet been fully uncovered. Since CD14 and CD16 expressions were first used to identify different monocyte subsets [20], several reports described a clinically relevant contribution of specific subsets to inflammation and repair, in noncardiac (e.g., asthma [21], infection by the human immunodeficiency virus [22]), and cardiovascular diseases (e.g., AMI and atherosclerosis). The involvement of specific monocyte subsets in tissue repair after AMI and the correct timing of their recruitment to the myocardium, which occurs in two phases, have recently been demonstrated to be critical for successful healing and regaining of normal function, both in humans [16] and mice [17]. After AMI, proinflammatory CD14^++^CD16^−^ monocytes (or their Ly6C^high^ mouse equivalents) are recruited to the damaged tissue by proinflammatory cytokines and chemokines (e.g., TNF*α*, IL-1*β*, and IL-6) during the first phase which lasts about 4 days. This monocyte subset is responsible for the removal of apoptotic myocytes, inflammatory cells, and necrotic cellular debris by phagocytosis, and for the release of proteases (e.g., MMPs, cathepsins, and urokinase plasminogen activator) that degrade the extracellular matrix and facilitate cell movement. The second phase depends on CD16^+^ monocytes or their Ly6C^low^ mouse equivalents (with no clear distinction between human CD14^+^CD16^+^ and CD14^dim^CD16^+^ monocytes), which promote angiogenesis through the secretion of VEGF and FGF, recruit myofibroblasts, and deposit collagen and other ECM proteins to form granulation and scar tissues. Thus, insufficient or exaggerated presence of monocyte in the heart, during the first or second phases, may contribute to impaired healing after myocardial damage, leading to myocardial remodelling and eventually to HF [23]. 

To the best of our knowledge, only two previous studies described changes in the distribution of monocytic subsets in HF patients, with conflicting results. Our finding of increased levels of the nonclassical CD14^dim^CD16^+^ and reduced levels of the classical CD14^++^CD16^−^ monocyte subsets in the peripheral blood of HF patients relative to healthy controls is consistent with one of the studies [18], but contradicts the other [24] that demonstrated the expansion of the CD14^+^CD16^+^ in HF patients, rather than the CD14^dim^CD16^+^ subset. This indicates that the role monocytes play during HF is only beginning to be explored and that the two subsets that make up the CD16^+^ monocytes population are not homogenous and may have different roles. The possible role of CD16^+^ monocytes was scarcely studied, and only few studies showed the secretion of proinflammatory cytokines from these monocytes, mostly in sepsis [25] or viral stimulation [14]. To the best of our knowledge, the role that CD14^dim^CD16^+^ subsets play in HF was not yet evaluated. HF is associated with inflammation, endothelial dysfunction, and oxidative stress [26, 27]. However, the inflammatory versus anti-inflammatory roles of both CD16^+^ monocytes (including CD14^dim^CD16^+^) and the IL-13 cytokine are highly controversial. We observed in our patients significantly increased levels of CD14^dim^CD16^+^ and reduced levels of CD14^++^CD16^−^. These CD14^dim^CD16^+^ cells, a part of the CD16^+^ population (Ly6C^low^ in mice), were associated with beneficial wound healing and decreased adverse remodelling processes in both human and animal models [17, 23]. Moreover, in contrast to CD14^++^CD16^−^, CD14^dim^CD16^+^ were shown to express less PSGL-1 and higher CX_3_CR1 [18]. PSGL-1 is associated with inflammation and endothelial dysfunction in atherosclerosis [28], which could be prevented by its deficiency [29]. CD14^dim^CD16^+^, as a part of the CD16^+^ cells, express higher CX_3_CR1 than CD14^++^CD16^−^ cells, which is associated with wound healing and limitation of oxidative stress and inflammation [30, 31]. Thus, the expanded CD14^dim^CD16^+^ subset can potentially reduce inflammation and endothelial dysfunction. We have further shown that HF patients have increased levels of IL-13 and that this subset can produce the cytokine. IL-13 by itself has anti-inflammatory properties [32, 33]. Collectively, it seems that the increase in CD14^dim^CD16^+^ subset combined with its ability to produce IL-13 may act as a counterbalance mechanism, designed to slow down the active remodelling process. This may explain the inverse correlation between the increased CD14^dim^CD16^+^ subset and reduced left heart dilatation as manifested in smaller EDD measurements. Of note, the CD14^++^CD16^−^ subset had no additional impact on the protective effect of CD^dim^CD16^+^ cells on the heart size.

Interestingly, previous results [24] showed inverse correlation between the expansion of the CD14^+^CD16^+^ subset and left ventricular ejection fraction (LVEF) in HF patients. This is compatible with our notion that the two CD16^+^ monocytic subsets may participate in the remodelling process in HF, either to enhance or limit it. The lack of correlation between the CD14^dim^CD16^+^ subset and LVEF or NYHA in our study may be explained by the relative homogeneous patient population that we recruited to our study, which consisted only of symptomatic, systolic HF patients defined as low LVEF (NYHA 2–4 and LVEF < 40%, resp.) [34].

Monocytes exert their effects partly through the secretion of both pro- and anti-inflammatory cytokines and chemokines, and elevated levels of these were described in HF sera previously [5, 6]. The role of some of these cytokines and chemokines in the development of HF has been described in details elsewhere [35–37]. In accordance with previous studies, we found elevated levels of TNF*α*, IL-1*β*, IL-10, and IL-13 in serum of HF patients [38–41], whereas levels of TGF*β* remained unchanged. We showed a significant positive correlation between CD14^dim^CD16^+^ and IL-13, suggesting a causal link between this anti-inflammatory cytokine and the monocyte subset. To further explore this premise, we stained the monocyte subsets for intracellular expression of IL-13 and demonstrated directly that the nonclassical subset can produce IL-13 in HF patients, but not in healthy controls. This finding is in agreement with a recent study that also showed increased plasma levels of IL-13 in HF patients, but did not link IL-13 levels to the expansion of one specific monocytic subset. The role of IL-13 in HF was hardly investigated, and only one study showed a negative correlation between IL-13 levels and LVEF values [39]. Furthermore, IL-13 knockout mice exhibit severe cardiomyopathy, impaired cardiac function, and HF [42]. These data imply that although IL-13 is a known mediator of tissue fibrosis and remodelling in several diseases [43, 44], its role in the remodelling process in HF may be more complex and even protective.

To the best of our knowledge, our study is the first to suggest that IL-13 production may be directly linked to the specific CD14^dim^CD16^+^ monocyte subset in HF patients. We suggest that this cytokine and the nonclassical CD14^dim^CD16^−^ monocyte subset that produces it may be important in the counterbalance systems aiming to slow the remodelling process which is so central in the pathogenesis of HF. This premise, however, requires further experimental support and merits further investigation. The hallmark of HF pathogenesis is the remodeling process. Thus, interference with the process of remodeling could potentially affect HF disease onset and progression. Our research now identifies two new targets, the CD14^dim^CD16^+^ subset and the IL-13 cytokine they produce, whose manipulation could potentially slow down the remodeling process and its deleterious clinical consequences. In addition, these two parameters (i.e., CD14^dim^CD16^+^ levels and IL-13 concentrations) may be used as novel biomarkers for HF patients' restratification according to the severity of their disease. However, all these potential clinical implications require further investigation.

## Figures and Tables

**Table 1:** Clinical characteristics of heart failure patients and controls.

Clinical characteristics	HF patients (*n* = 59)	Control (*n* = 29)
Age (years; mean ± sd)	58.1 ± 13.9	59.7 ± 6.4
Sex (male/female)	45/14	15/14
NYHA 1	7	N/A
NYHA 2	39	N/A
NYHA 3	11	N/A
NYHA 4	2	N/A
Ischemic etiology	30 (51%)	N/A
Diabetes mellitus	21 (35.6%)	4 (14%)
*β*-blockers (*n*/%)	59 (100%)	2 (7%)
ACE-I +/or ARB (*n*/%)	56 (95%)	2 (7%)
Aldosterone antagonist (*n*/%)	18 (30.5%)	None
Statins (*n*/%)	44 (75%)	N/A
LV ejection fraction (%, SD)	26.29 ± 8.63	N/A
Mean hemoglobin (g/dL, mean ± sd)	*12.9 ± 1.8 *	*N/A *
Mean creatinine (mg/dL, mean ± sd)	*1.18 ± 0.06 *	*N/A *
Creatinine clearance (CCT, mean ± sd)	*81 ± 4.4 cm/min *	*N/A *

**Table 2:** Association between the three monocyte subsets and HF.

Monocyte subset	Control mean ± SD (median)	HF patients mean ± SD (median)	O.R.^a^	95% CI	*P* value
CD14^++^CD16^−^	84.3 ± 10.63 (86.9)	73.5 ± 14.5 (77.4)	0.894	0.834–0.958	0.001***
CD14^+^CD16^+^	3.9 ± 4.11 (2.6)	3.6 ± 4.3 (2.5)	1.008	0.902–1.125	0.891^ns^
CD14^dim^CD16^+^	6.5 ± 5.3 (5.3)	9.3 ± 4.0 (9.3)	1.179	1.038–1.339	0.011*

^a^Each odds ratio calculated by the logistic regression model is adjusted for age and gender.

^∗,∗∗,∗∗∗^Significance, ^ns^non-significance.

**Table 3:** Association between the CD14^dim^CD16^+^ monocytic subset and parameters of HF severity.

Parameter	Cutoff value	CD14^dim^CD16^+^ ≤ 7.8 *N*, %	CD14^dim^CD16^+^ > 7.8 *N*, %	O.R.	95% CI	*P* value
EDD	<6 ≥6	6, 26.1% 17, 73.9%	21, 58.3% 15, 41.7%	0.2521	0.0804–0.790	0.015*
LVEF	<25 ≥25	9, 15.2% 14, 23.7%	13, 22.0% 23, 39.0%	1.137	0.3866–3.346	0.8151
NYHA	<2 ≥2	2, 3.3% 21, 35.6%	5, 8.5% 31, 52.5%	0.5905	0.1045–3.335	0.5474

**Table 4:** Association between serum cytokines and HF.

Cytokine	Cutoff value	Control *N*, (%)	Range (pg/mL)	HF patients *N*, (%)	Range (pg/mL)	O.R.^a^	95% CI	*P* value	AUC (95% CI)
TNF*α* (pg/mL)	<0 ≥1	20, 87% 3, 13%	0–2.7	30, 61% 19, 39%	0–276	4.175	1.045–16.68	0.043*	0.720 (0.597–0.843)
IL-1*β* (pg/mL)	<9.8 ≥9.8	16, 70% 7, 30%	0–51	21, 41% 30, 59%	0–362	3.390	1.133–10.14	0.029*	0.692 (0.573–0.811)
IL-10 (pg/mL)	<10 ≥10	16, 70% 7, 30%	0–18.9	20, 41% 29, 59%	0–236	3.751	1.237–11.38	0.020*	0.733 (0.609–0.857)
TGF*β* (pg/mL)	<100 ≥100	10, 43% 13, 56%	28–280	27, 53% 24, 47%	27–619	0.694	0.252–1.91	0.479^ns^	0.657 (0.527–0.788)
IL-13 (pg/mL)	<9 ≥9	20, 87% 3, 13%	0–34	17, 33% 34, 64%	0–371	14.393	3.48–59.5	<0.001***	0.797 (0.685–0.908)

^a^Odds ratio calculated by the logistic regression models is adjusted for age and gender.

**Table 5:** Correlation between the CD14^dim^CD16^+^ and CD14^++^CD16^−^ monocytic subsets and serum cytokines in controls and HF patients.

	TNF*α*	IL-1*β*	IL-10	TGF*β*	IL-13
CD14^++^CD16^−^					
*r *	−0.307	−0.370	0.019	−0.104	−0.412
*P *	0.009**	0.001***	0.875^ns^	0.377^ns^	<0.0001***
CD14^dim^CD16^+^					
*r *	−0.037	−0.085	−0.059	−0.096	0.277
*P *	0.756^ns^	0.472^ns^	0.622^ns^	0.418^ns^	0.017*
